# Isoelectric Point of Proteins at Hydrophobic Interfaces

**DOI:** 10.3389/fchem.2021.712978

**Published:** 2021-07-30

**Authors:** Vanessa Lautenbach, Saman Hosseinpour, Wolfgang Peukert

**Affiliations:** Institute of Particle Technology (LFG), Friedrich-Alexander Universität Erlangen-Nürnberg (FAU), Erlangen, Germany

**Keywords:** proteins, isoelectric point, sum frequency generation spectroscopy, surface hydrophobicity, zeta potential

## Abstract

Structural and colloidal stability of proteins at different surfaces and interfaces is of great importance in many fields including medical, pharmaceutical, or material science. Due to their flexibility, proteins tend to respond to their environmental conditions and can undergo structural and conformational changes. For instance, alterations in physiological factors such as temperature, ions concentration, or pH as well as the adsorption to an interface can initiate protein aggregation. Therefore, at different surfaces and interfaces the characterization of the structural and colloidal stability of proteins, which is mainly influenced by their electrostatic and hydrophobic interactions, is of fundamental importance. In this study, we utilized sum frequency generation (SFG) spectroscopy to assess the role of solution pH on the polarity and magnitude of the electric field within the hydration shell of selected model proteins adsorbed to a hydrophobic surface. We used polystyrene (PS) as a model hydrophobic surface and determined the isoelectric point (IEP) of four structurally different model proteins. Comparing the measured IEP of proteins at the PS/solution or air/solution interface with that determined in the bulk solution *via* zeta potential measurement, we found significant similarities between the IEP of surface adsorbed proteins and those in the bulk aqueous phase. The pH dependence behavior of proteins was correlated to their amino acid composition and degree of hydrophobicity.

## Introduction

Proteins are flexible macromolecules that react sensitively to environmental conditions and external stimulators. For instance, alteration in physiological factors such as temperature, ion concentration, or pH often results in changes in the secondary or tertiary structure of proteins, their solubility, or colloidal stability (Sarkar et al., 2009; Maldonado-Valderrama et al., 2010). Moreover, the adsorption of proteins to different surfaces, e.g., cell membranes and implants in biological systems or pipings during industrial processing, is often accompanied by proteins’ structural and conformational alterations (Yano, 2012; FaulónMarruecos et al., 2018; Mitra, 2020). These changes in protein structure not only affect protein functionality but also may trigger their abnormal folding. For instance, the hydrophobicity of cell membranes can initiate protein aggregation, which is assumed to be the cause of severe neuronal diseases such as Alzheimer’s or Parkinson’s disease (Beyer, 2007; Gonzalez-Garcia et al., 2021; Saghir et al., 2021). Similarly, in downstream processing and adsorption chromatography of proteins irreversible binding of proteins on commercially available hydrophobic adsorbents is accompanied by structural changes in proteins, a process that is influenced by solution pH (Millitzer et al., 2005).

Hence, molecular understanding of the protein behavior at different surfaces and interfaces is of great importance in many fields including biopharmaceutical development, drug targeting, development of implant materials, or biomembrane processing. So far numerous experimental and computational studies have been performed on model proteins such as serum albumin, beta-lactoglobulin, or lysozyme to unravel their specific interactions with different surfaces. Among many parameters that affect protein adsorption, conformation, and stability at different surfaces and interfaces, hydrophobic and electrostatic interactions are considered to be the main factors (van Dulm and Norde, 1983; Norde, 1996; McUmber et al., 2015). Upon interaction with a surface, proteins usually adopt a different conformation than those in the bulk solution, to minimize their free energy. For instance, Roach et al. (2005) showed that increased surface hydrophobicity increases the adsorption affinity of amphiphilic proteins and triggers structural changes. Meanwhile, electrostatic attraction or repulsion not only affect the adsorption of proteins on surfaces but also alters proteins’ structural and conformational stability. The magnitude of electrostatic interactions between proteins and surfaces and between protein molecules can be altered either by the ionic content or the pH value of the surrounding medium. At pH values close to the isoelectric point (IEP), proteins possess a net neutral charge whereas at pH values above and below IEP they exhibit net negative and net positive charge, respectively. The IEP of proteins is strongly influenced by the composition of amino acids, their local distribution in protein structure, as well as the structural conformation of proteins. The latter, as was mentioned earlier, is dependent on the surfaces to which proteins adsorb.

In this work, we address the question of whether the adsorption of proteins at a hydrophobic solid surface leads to a change in electrostatic interactions and consequently to a shift in the isoelectric point of proteins. As previously mentioned, the adsorption as well as the structural and conformational stability of proteins are affected by important factors like electrostatic forces, the structure and dynamics of the hydration layer surrounding proteins, and hydrophobic interactions. However, characterization of protein properties at surfaces and interfaces is experimentally challenging and requires surface sensitive analytical tools to differentiate the overwhelming number of molecules in the bulk phase from those at surfaces and interfaces. Over the last decades, sum frequency generation (SFG) spectroscopy has proven to be a powerful tool for this purpose and SFG has been applied to study proteins at interfaces in numerous cases (Dreesen et al., 2004; Vidal and Tadjeddine, 2005; Zhang et al., 2013; Hosseinpour et al., 2020).

In this work, we present the SFG results of four proteins at the hydrophobic polystyrene (PS)/water interface to assess the polarity and magnitude of the electric field within the hydration shell of the adsorbed proteins on a solid hydrophobic surface. Accordingly, the IEP of each protein was precisely determined at the buried PS/solution interface, by analyzing the SFG spectra of each protein as a function of solution pH. Based on the previous studies on the IEP of proteins in the bulk solutions and at the liquid/air interface, in this study we have selected model proteins with very different hydrophobicity indices and bulk isoelectric points, as these parameters are expected to have the most dominant impacts on protein adsorption and restructuring at hydrophobic surfaces and interfaces.

The comparison between the measured IEP of proteins at the buried PS/solution interface and at the air/solution interface (Guckeisen et al., 2019) as well as with the IEP measured in the bulk solution *via* zeta potential showed significant similarities between the IEP of proteins in the bulk aqueous phase and those adsorbed at the air/liquid or solid/liquid interface.

## Materials and Methods

### Protein Sample Preparation

For the determination of the IEP at the solid/liquid interface, four proteins were chosen, which differ in terms of their bulk IEP and hydrophobicity (see Table 1). Bovine serum albumin (BSA, A7030), hemoglobin (H7379), and lysozyme (L6876) were purchased from Sigma-Aldrich (St. Louis, Missouri, United States). Antifreeze protein type III (AFP III) was obtained from A/F Protein Inc., Waltham, United States. The proteins were used as received from the manufacturer with no further purification and protein solutions in concentrations of either 0.1 g/L, 1 g/L, or 2 g/L were prepared. The protein concentrations that are used are all below the solubility limit of the corresponding proteins (see Table 1). To keep the ionic strength constant, the desired amount of protein lyophilisate was dissolved in 10 mM NaCl (≥99.8%, Carl Roth GmbH, Karlsruhe, Germany) solution. A QUINTIX64-1S analytical lab balance with the measurement accuracy of 0.1 mg from Sartorius AG (Goettingen, Germany) was used. For each protein, different pH values were set between 1.7 and 11.3 by adding HCl (0.1 M Honeywell™ Fluka™, Thermo Fisher Scientific Inc., Schwerte Germany) and NaOH (0.1 M Merck KGaA, Darmstadt, Germany) to the solution. The pH was determined by a inoLab pH7110 pH meter from WTW (Xylem Analytics Germany Sales GmbH and Co. KG, Weilheim, Germany) equipped with an InLab-Micro-Pro-ISM pH electrode from Mettler Toledo GmbH (Greifensee, Switzerland).

**TABLE 1:** Different Protein properties and comparison of different IEP.

Protein property	BSA	Lysozyme	Hemoglobin	AFP III
UniPort ID	P02769	P00698	P69905/P68871	P12416
Molecular weight/kDa	66.5	14.4	64.5	9.4
α-helix content/%	47	20	56	-
β-sheet content/%	0	10	0	-
Solubility in water/mg ml−1	40^a^	10^a^	20^a^	20^b^
Aromatic amino acid content/%	8.4	9.3	8.4	1.5
Hydrophobicity index^c^	−0.43	−0.15	0.03	0.41
Theoretical IEP^d^	5.59^e^	8.37^e^	7.29	8.94
Bulk IEP (zeta potential)	5.1	10	7.1	6–8
Air/solution IEP (SFG)	5.5	7–9.5	6.5	8
dPS/solution IEP (SFG)^f^	5.5	8.3	7.0	6–8

a Sigma Aldrich data sheets and

b manufacturers website.

c Hydrophobicity was calculated with GPMAW lite (Hoejrup).

d The theoretical IEPs were determined with an isoelectric point calculator (Kozlowski, 2016) from the proteins amino acid sequence given by the listed UniPort accession number.

e These calculated theoretical IEP values differ from the previously published ones (Guckeisen et al., 2019), because of the usage of different protein amino acid sequences.

f The uncertainties in the determination of the IEPs of the studied proteins include: the minor inaccuracy in protein concentration in the prepared solutions, the minor changes in the solution pH during data collection, the temporal fluctuations in the intensity of the IR and Vis beams during the SFG measurements, and the contribution of spectral noise in the determination of the minimum signal intensity. Attempts are made to minimize these possible inaccuracies during our measurements.

The solubility values were obtained by the proteins

### Prism Coating with Polystyrene

For the investigation of the proteins on the hydrophobic surface, deuterated polystyrene (dPS, PolymereSource Inc., Dorval, Canada) was spin coated on a CaF_2_ dove prism (Ma Teck GmbH, Jülich, Germany), following the procedure used by Wang et al. (2003). Before each coating, the prism was ultrasonically cleaned in toluene (≥99.5% VWR, Darmstadt, Germany), ethanol (≥99.8%, VWR, Darmstadt, Germany), and Alconox® (Alconox Inc., NY, United States) solution for 15 min each and finally rinsed with water. After this procedure, the prism was treated for 5 min in an O_2_ plasma oven (Femto, Diener electronic GmbH + Co. KG, Ebhausen, Germany) in order to remove persistent contaminations from the surface. A stock solution of 2 w% dPS in deuterated toluene (≥99.00%, Sigma-Aldrich, St. Louis, Missouri, United States) was used to coat the prism. After fixing the prism on the spin coater (KLM Spin-Coater SCC, Schaefer Technologie GmbH, Langen, Germany) using a home built holder, 100 µl of dPS solution was added on the large flat side of the cleaned prism. The prism was rotated at 20 rotations per second for 5 s and the coated prism was dried overnight at room temperature. The cleanliness of the dPS layer was controlled by measuring the SFG response at the dPS-air interface in the C-H stretching spectral region.

### Sum Frequency Generation Measurements

**FIGURE 1:**
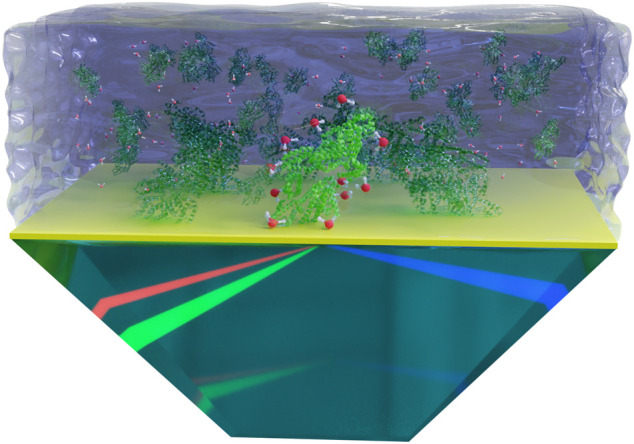
A schematic representation of the SFG measurements in the TIR geometry. The dPS layer is depicted in yellow. Proteins and water molecules are depicted out of scale for better visualization. Note that the preferential adsorption sites and the possible rearrangement of proteins upon adsorption to the hydrophobic surface are not considered in this schematic figure.

For the determination of the proteins’ IEP at the interface, SFG spectra were obtained by a broadband SFG spectrometer, which is described in detail elsewhere (Beierlein et al., 2015; Braunschweig et al., 2016). The spatial and temporal overlap of an etalon-narrowed beam in the visible wavelength range [ω_vis_ = 800 nm, full width half maximum (FWHM) ≈ 10 cm^−1^] and a femtosecond infrared beam (ω_IR_, FWHM ≈ 200 cm^−1^) generates an SFG signal (ω_SFG_ = ω_vis_ + ω_IR_). The IR wavelength was tuned in the frequency range of 2,800–3,800 cm^−1^ in five steps with 20 s acquisition time per step and accumulations of five for each spectrum. The SFG spectra of proteins on the surface of the dPS coated prism were collected at the total internal reflection (TIR) geometry (See Figure 1), enhancing the signal-to-noise ratio (Zhang et al., 2015). The dPS coated prism was fixed in a home built cell, which allows the change of the height, lateral position, and tilt of the sample surface with high precision. The height of the sample surface was controlled *via* a laser height sensor (LK-H052, Keyence Corporation, Osaka, Japan). For each protein, a set of different pH solutions were placed successively on top of the dPS coated CaF_2_ prism. In order to exclude signal deviations due to the possible inhomogeneities in the dPS layer, the series of measurements for one protein were carried out on the same coated prism without changing the spot of the laser irradiation (i.e., without moving the sample). The intensity of the incoming laser beams and the duration of the signal acquisition were adjusted to ensure no laser induced sample damaging occurred during the measurements. All SFG measurements were performed in the SSP (S-polarized SFG signal, S-polarized visible beam, and P-polarized IR beam) polarization combination where S stands for perpendicular and P for parallel to the plane of incidence.

### Zeta Potential Measurements

For the determination of the proteins’ IEP in the bulk phase, the zeta potential for all protein solutions was measured with a Nano ZS Zetasizer instrument (Malvern Instruments, Herrenberg, Germany), in which a folded-capillary cuvette (Malvern Instruments, Malvern, Grovewood, United Kingdom) was used. The temperature was controlled at 25°C and the initial equilibration time was set to 120 s. The zeta potential is correlated with the proteins’ net surface charge (Keppler et al., 2021), hence, the pH value for which the measured zeta potential value reaches 0 mV was determined as the bulk IEP of the corresponding protein. Each sample was measured three times within one measurement cycle and the results were averaged.

## Results and Discussion

In this study, the IEP of four proteins BSA, lysozyme, AFP III, and hemoglobin were investigated in contact with a hydrophobic layer at the dPS-water interface, using SFG spectroscopy. The dPS layer represents an exemplary hydrophobic surface on which the proteins adsorb and react to the different solution pH values. As presented in Table 1, these proteins differ in terms of their IEP in the bulk and their hydrophobicity index, which are the main factors affecting their electrostatic and hydrophobic interactions, respectively. Other important characteristics of the studied proteins such as molecular weights and solubility as well as the content of α-helix and β-sheet in their structures are also provided in Table 1.

Based on the SFG spectroscopy selection rules, to obtain an SFG signal, molecules should reside in a non-centrosymmetric environment, must have a certain degree of order, and should contain vibrations that are simultaneously IR and Raman active. These selection rules provide SFG with an inherent surface sensitivity, which allows detecting surface adsorbed molecules without the inclusion of bulk molecules in the signal.

As demonstrated in Eq. 1 the SFG signal intensity is a function of non-resonant NR and resonant R parts of the second-order nonlinear electric susceptibility ${\text{χ}}^{\left(2\right)}$ and is proportional to the intensities of the fundamental incoming visible $I_{vis}$ and infrared $I_{IR}$ beams. (Richmond, 2002; Shen and Ostroverkhov, 2006; Zhang et al., 2013).

$${\text{I}}_{\text{SFG}}\propto \;{\left|{\text{χ}}_{NR}^{\left(2\right)}+{\text{χ}}_{R}^{\left(2\right)}\right|}^{2}.\;I_{vis}.I_{IR}\;\propto \;{\left|{\text{χ}}_{NR}^{\left(2\right)}+\sum_{n}\frac{A_{n}}{\omega_{n}-\omega_{IR}+i{\text{Γ}}_{n}}\right|}^{2}.\;I_{vis}.I_{IR}$$ (1)

The non-resonant portion of the SFG signal is frequency independent and the resonant part can be described as a function of the amplitude $A_{n}$, damping factor ${\text{Γ}}_{n},$ and the frequencies of the IR ($\omega_{IR}$) and the *n*th vibrational mode ($\omega_{n}$).

The mathematical relation between the oscillator strength $A_{n}$ and the number N of contributing oscillators in Eq. 2 further shows that the SFG signal intensity also scales with the number of ordered molecules at the surface.

$$A_{n}=Na_{n}$$ (2)

in which the angular brackets refer to an orientational average over all interfacial molecules and $a_{n}$ describes the molecules’ tensorial mode strength (Shen and Ostroverkhov, 2006). At the electrified surfaces, the interfacial potential (Φ(0)) interacts with both ${\text{χ}}^{\left(2\right)}$ and third-order susceptibility $\left({\text{χ}}^{\left(3\right)}\right)$. However, for comparison of the SFG results with those published earlier (Guckeisen et al., 2019), here we make no distinction between the relative contribution of ${\text{χ}}^{\left(2\right)}$ and ${\text{χ}}^{\left(3\right)}$ and use effective ${\text{χ}}^{\left(2\right)}$ for fitting our SFG spectra, as was utilized by Das et al. (Das et al., 2019).

Proteins at pH values above and below their corresponding IEP become negatively or positively charged, respectively. Accordingly, the polar water molecules surrounding proteins adopt a preferential H-up and H-down configuration, with respect to the protein surface. The larger electric field (i.e., proteins with higher net charge) hence aligns a greater number of water molecules with the same orientation resulting in an enhanced SFG signal (see Eq. 2).

**FIGURE 2:**
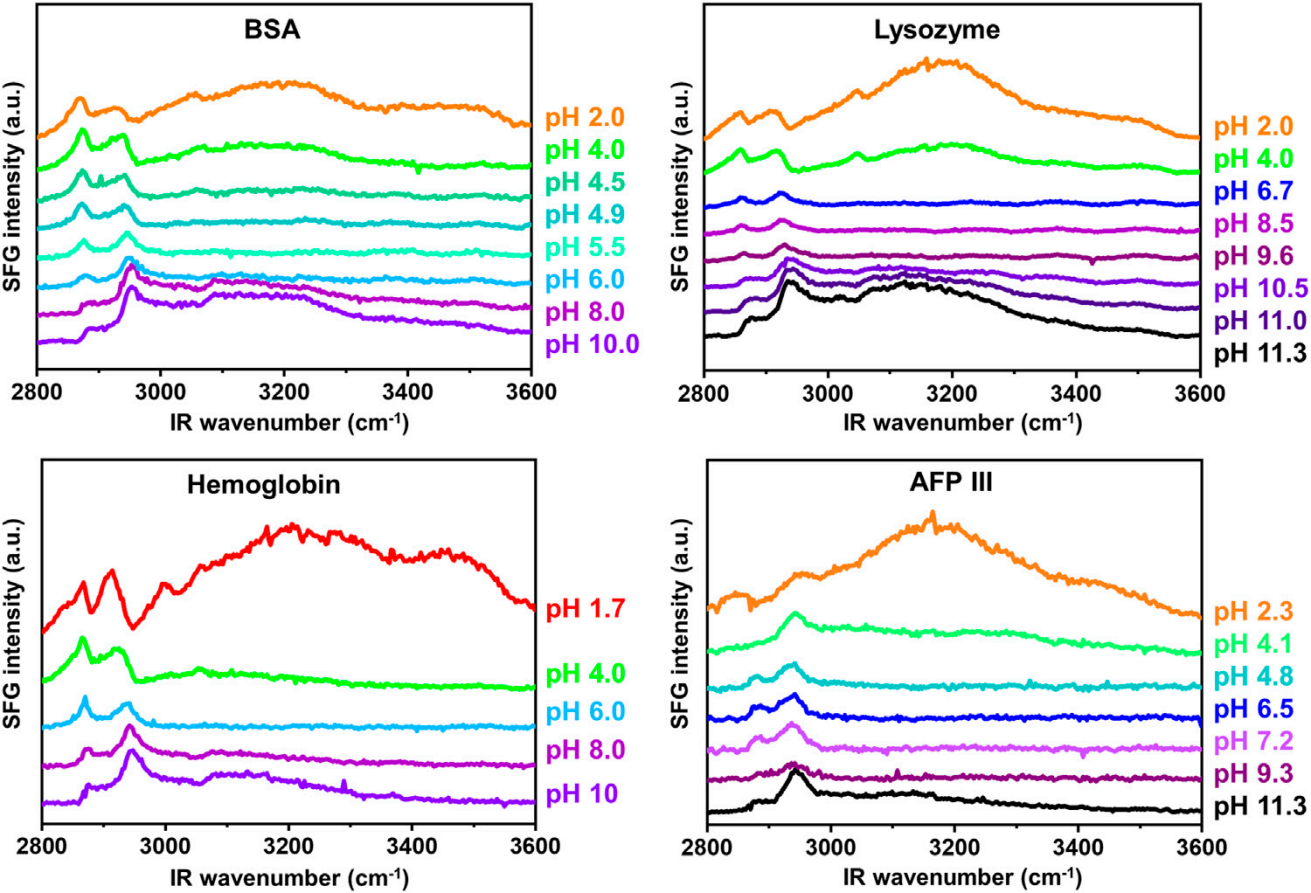
pH dependent SFG spectra measured in ssp polarization combination in the wavenumber range of 2,800cm^−1^ and 3,600 cm^−1^.

Figure 2 shows the recorded SFG spectra of the investigated proteins as a function of the solution pH over a wavenumber range from 2,800 cm^−1 ^to 3,600 cm^−1^. Multiple vibrational modes can be recognized in this spectral region. Within the interval from 2,800 cm^−1^ to 3,000 cm^−1^, the CH vibrations from the proteins side chain amino acids appear, the assignments of which have been provided elsewhere (Chen et al., 2007).

Another peak can be detected at ∼3,060 cm^−1^, which represents the so-called ring mode and originates from vibrations of the aromatic rings in amino acids such as phenylalanine, tryptophan, and tyrosine. This particular peak is expressed clearly for BSA, lysozyme, and hemoglobin, especially in predominantly acidic or basic pH regimes.

With increasing the solution pH, the ring mode peak undergoes a sign change (from a positive to a negative peak) and its amplitude passes zero during this transition. This observation is consistent with previous publications (Engelhardt et al., 2013; Guckeisen et al., 2019; Wang et al., 2002) and is a consequence of the interference between the proteins’ ring mode vibration and interfacial water, which can be either constructive or destructive depending on the proteins’ net charge (Guckeisen et al., 2019). The transition point of the sign of the ring mode peak is a first approximation for determining the IEP at the interface since the IEP describes the state at which the protein is net uncharged. The measured SFG spectra for AFP III, as demonstrated in Figure 2, do not show significant peak intensity of the ring mode over the whole pH range. Referring to Table 1, it becomes evident that the negligible ring mode SFG signal in the AFP III spectra correlated well with its relatively low content of aromatic amino acids. It should, nevertheless, be noted that the absolute orientation of the ring mode in the structure of proteins would also affect the intensity of the corresponding peaks in their SFG spectra (Naseri et al., 2018).

In the frequency region of 3,100 cm^−1^ to 3,600 cm^−1^, broad vibrational modes are observed from the OH stretching vibrations of polar water molecules, which arrange themselves at the buried dPS/solution interface due to the charge state of the protein (Ohshima, 1995; Po and Senozan, 2001; Beierlein et al., 2015). As can be seen in Figure 2, the signal intensity in this frequency region is very sensitive to the change of the solution pH. According to Eqs 1, 2, the SFG signal intensity at this frequency region is mainly influenced by the number density of the ordered interfacial molecules, whose extent of order can be triggered by the pH dependent charge of the proteins. In other words, the higher the absolute charge of proteins, the higher the intensity of the OH SFG signal intensity, because more polar water molecules similarly oriented at the buried dPS/solution interface. At extreme acidic solutions, in which the proteins are predominantly protonated and positively charged, the OH signal intensity reaches a maximum since a large number of water molecules are aligned with oxygen toward the protein at the buried dPS/solution interface. With increasing pH, the OH signal intensity first approaches a minimum and then reaches another maximum at extreme alkaline solutions.

**FIGURE 3:**
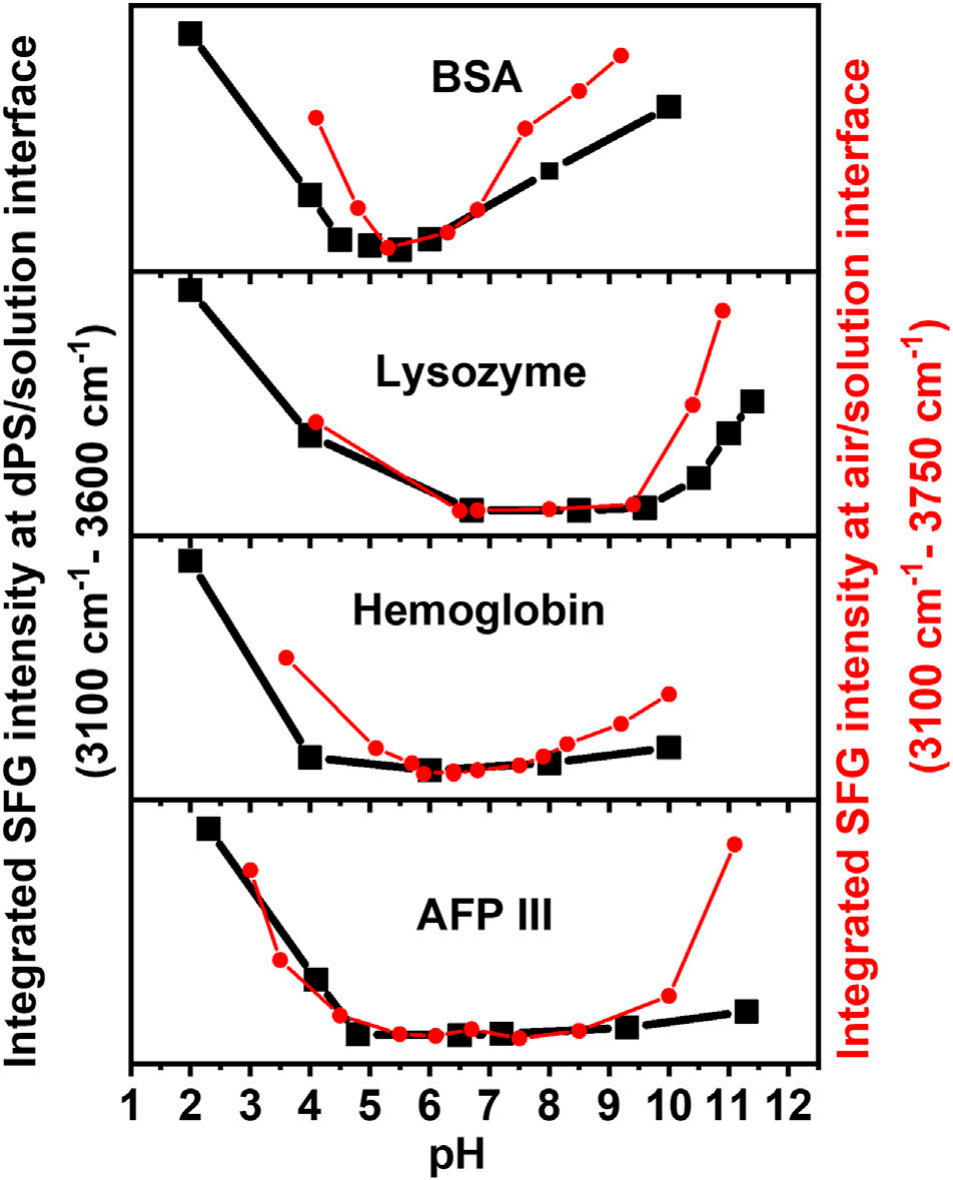
Integrated SFG signal intensity in the range between 3,100 cm^−1^ and 3,600 cm^−1^ (at the buried dPS/solution interface, black) and between 3,100 cm^−1^ and 3750 cm^−1^ (at the air/solution interface, red) for four different proteins. The latter data are reproduced from (Guckeisen et al. (2019)). Adapted with permission from Langmuir 35 (14), 5004–5012. Copyright 2021 American Chemical Society.

To precisely determine the isoelectric point at the buried dPS/solution interface, the SFG spectra were integrated in the range of 3,100 cm^−1^ and 3,600 cm^−1^ and the results are provided in Figure 3. For all proteins, the integrated SFG signal intensity passes through a global minimum, which represents the IEP of the corresponding protein at the buried dPS/solution interface. For comparison, the pH dependent integrated SFG signal intensity of the same proteins at the air/solution interface is also shown in Figure 3 [red curves, right *Y*-axis, reproduced from Guckeisen et al. (2019)]. As also tabulated in Table 1, a very good agreement exists between the measured IEPs of proteins at the dPS/solution interface and those at the air/solution interface. Interestingly, the measured IEPs of the surface adsorbed proteins (at either dPS/solution or the air/solution interfaces) do not differ from the corresponding theoretically calculated or measured IEPs *via* Zeta potential measurements (*vide infra*), which denotes that the electrostatic interactions in the studied proteins are independent of their local environment or the interface to which they adsorb.

Besides the general agreement in the IEPs of the surface adsorbed proteins, there are other similarities and some differences in the observed trends in the integrated SFG signal intensity of the proteins at the dPS/solution and the air/solution interfaces (Figure 3, black and red curves). To some extent, the changes in the integrated SFG signal intensity above and below the IEP of the investigated proteins are asymmetrical, to some extent. Furthermore, the broadness of the region corresponding to the minimum integrated SFG signal (hereafter referred to as “minimum region”) differs for different proteins; it is narrowest for BSA and broadest for AFP III at both the dPS/solution and the air/solution interfaces. The asymmetry of the curves and width of this “minimum region” in Figure 3 correlates with the dissociation constant (pK_a_) values of the individual ionizable amino acids in the proteins’ primary structure and their structural stability. According to Pace et al., glutamate (Glu), aspartate (Asp), cysteine (Cys), tyrosine (Thr), histidine (His), lysine (Lys), and arginine (Arg) are the predominant ionizable amino acids, which with the exception of cysteine, all have a negative hydrophobicity index and thus belong to the polar amino acids. These amino acids are mainly located in the hydrophilic regions of the protein and are most likely to be in contact with the polar water (Pace et al., 2009; Kozlowski, 2016), as will be discussed in the following. Comparing the results in Table 1 and Figure 3, the role of the amino acid composition of proteins and their hydrophobicity in determining the width of the “minimum region” becomes evident. Narrower “minimum regions” are observed for the proteins in which the pK_a_ of their constituent amino acids are close to each other. Moreover, proteins with a large negative hydrophobicity index (such as BSA) are composed of a higher proportion of polar amino acids and have a more symmetrical and narrower “minimum region.” However, it is noticeable that the black curves (i.e., proteins at the buried dPS/solution interface) in Figure 3 have a slightly flatter “minimum region” compared to the red curves (i.e., proteins at the air/solution interface), a phenomenon which is related to the less hydrophobic nature of air compared to dPS. A possible explanation for this behaviour could be the more pronounced conformational change in the proteins’ structure after the adsorption to the dPS/solution interface. Upon adsorption to a surface, proteins tend to undergo structural changes to minimize their free energy (ΔG). For instance, the adsorption free energy of proteins is reduced by the displacement of the water molecules surrounding the nonpolar amino acid residues that interact with the nonpolar solid surfaces (Rabe et al., 2011; Latour, 2020; Mitra, 2020). As described by Rabe et al. (2011) and Rahimi et al. (2021), depending on the electrostatic interactions between the adsorbing proteins and substrate, as well as the density of the surface, different pathways of adsorption (e.g., cooperative or non-cooperative) can be expected. It is also discussed that proteins adsorb more strongly to hydrophobic surfaces than to hydrophilic surfaces.

The difference between the black and red curves in Figure 3 is more dominant in the basic regime (especially for Lysozyme and AFP III), which is consistent with previous studies showing that proteins at the surface tend to undergo more structural changes in the basic pH regime than in the acidic solutions (Guckeisen et al., 2021).

The similarity between the IEP of the surface adsorbed proteins (either at the dPS/solution or at the air/solution interfaces) and those found in the bulk solution (see Table 1) can also be correlated to the role of hydrophobic side chain amino acids in proteins structure. As provided in this table, for each protein a theoretical hydrophobicity index can be calculated from the hydrophobicity of the individual amino acid constituents. Depending on the type and sequence of amino acids in a protein, they contribute with a negative or positive index to the total hydrophobicity index of the proteins. According to Kyte and Doolittle (1982), amino acids such as isoleucine and valine with a hydrophobicity index value of 4.5 and 4.2 have the highest hydrophobicity and arginine and lysine with the hydrophobicity index value −4.5 and −3.9 have the lowest hydrophobicity, respectively. Comparing the amino acid content in the structure of studied proteins, it became evident that BSA and Lysozyme contain a relatively small amount of hydrophobic amino acids, whereas hemoglobin and AFP III consist mainly out of the hydrophobic valine and are more hydrophobic. BSA and Lysozyme include more amino acids with negative hydrophobicity indices, which are preferably located in the hydrophilic part of the protein and therefore are more probable to come in contact with surrounding water molecules. Di Rienzo et al. developed a computational method to predict the hydrophobicity of amino acid side chains in proteins based on the orientation of surrounding water molecules. They defined four groups of amino acids by applying a principal component analysis and distinguish between negatively charged, positively charged, polar, and nonpolar amino acids. Accordingly, comparing the amino acid content of investigated proteins in this study, it is confirmed that hemoglobin and AFP III consists mainly out of hydrophobic amino acids whereas in BSA and Lysozyme most prevalent amino acids belong to the charged group (Di Rienzo et al., 2021).

In the bulk aqueous phase, hydrophobic amino acids of proteins are preferentially localized in the hydrophobic inner part of the protein, away from the surrounding water molecules (Janin, 1979). Similarly, at both dPS/solution interface and air/solution interface, primary interactions take place between the nonpolar parts of the protein and the hydrophobic interface. Therefore, the hydrophobic side chains of the surface adsorbed proteins protrude toward the hydrophobic phase (i.e., dPS or air, respectively), whereas the hydrophilic side chains face toward polar water molecules. Hence, these hydrophilic amino acids contribute the most to the electrostatic interactions with the surrounding medium or with other protein molecules, whereas the shielded hydrophobic amino acids (in the core of bulk proteins, or those protruding to hydrophobic dPS or air) do not come into the direct contact with water and have minor effects on the overall electrostatic interactions in protein systems. Indeed, reorientation of the proteins and the change in their secondary and tertiary structure are also plausible under these conditions to minimize the overall free energy of the system. Using coarse grain protein simulations, Zhao and Cieplak show that proteins are twisted at both air/solution and oil/solution interface and support the explanation for the equality of the IEPs given in this paper (Cieplak et al., 2014; Zhao and Cieplak, 2017).

## Conclusion

In this study, we utilized an inherently surface sensitive nonlinear spectroscopic tool, sum frequency generation (SFG) spectroscopy, to assess the impact of solution pH on selected model proteins adsorbed at a solid hydrophobic surface. Deuterated polystyrene (dPS) was used as a model hydrophobic surface and the changes in the polarity and magnitude of the induced electric field at the dPS/solution interface were utilized as a function of the solution pH to determine the IEP of surface adsorbed proteins.

The measured IEPs for proteins adsorbed to the dPS/solution interface were comparable to those at the air/solution interface and IEPs measured for proteins in the bulk solution. The IEP of proteins and the trends in the change of the electrostatic interactions in proteins as a function of solution pH were described based on the amino acid content and hydrophobicity of the studied proteins. Our results indicate that the IEP of proteins is mainly dependent on the polar amino acids in their structure, which are similarly accessible to surrounding water molecules in the bulk solution and at the PS/solution or air/solution interface, despite the possible reorganization of proteins and changes in their secondary structure upon adsorption to hydrophobic interfaces.

## Data Availability

The datasets presented in this study can be found in online repositories. The names of the repository/repositories and accession number(s) can be found below: https://www.uniprot.org/, BSA: P02769 Lsyozyme: P00698 Hemoglobin: P69905/P68871 AFP III: P12416.
